# Antibody drug conjugate, a level-up version of monoclonal antibody?

**DOI:** 10.1097/JS9.0000000000001748

**Published:** 2024-06-04

**Authors:** Yuqi Yang, Yue Zheng, Xu Sun, Ailin Zhao, Yijun Wu

**Affiliations:** a Division of Thoracic Tumor Multimodality Treatment, Cancer Center,West China Hospital, Sichuan University; b Department of Hematology West China Hospital, Sichuan University; c Laboratory of Clinical Cell Therapy, West China Hospital, Sichuan University; d West China School of Medicine, Sichuan University, Chengdu, Sichuan, People’s Republic of China

*Dear Editor*,

Due to its extremely high mortality rate, pancreatic cancer is still a challenge that researchers are eager to solve. Of these, pancreatic ductal adenocarcinoma (PDAC) is the most prevalent and aggressive, accounting for ~90% of pancreatic cancers. In recent years, more and more drug treatments for PDAC have emerged as research advances. This article, *Pancreatic Cancer: New Approaches to Drug Therapy* ^1^, in great detail, summarizes the different categories of drug treatments currently available in the field of PDAC. In addition to mentioning well-known chemotherapeutic agents, immunotherapies, and molecularly-targeted therapies, the article also describes the cutting-edge and promising DNA damage repair inhibitors, metabolic inhibition therapies (potential therapies targeting metabolic pathways specific to pancreatic cancer), and extracellular matrix-targeted therapies. All of these show multiple advances in the field of pancreatic cancer treatment and provide a very comprehensive summary of drug development in this area. However, with respect to the immunotherapeutic drugs mentioned, we would like to add some therapeutic information about the use of Antigen drug conjugates (ADCs) for the treatment of pancreatic cancer that was not mentioned in that article. We would also like to share our understanding of the relationship between ADCs and monoclonal antibodies, which are often the only approved immunotherapeutic drugs at present.

## Current status of ADC drugs

ADC, as we can easily tell from its name, consists of a monoclonal antibody, a linker and a cytotoxic drug. Last decade has already witnessed its marvelous advantages of highly specific targeting ability and potent killing effect towards cancer cells. By the end of 2023, 16 ADCs have been approved worldwide. Chemotherapy is currently the first-line of treatment for pancreatic cancer. However, it has limited effect on improving the survival rate of pancreatic cancer patients and is accompanied by strong side effects. ADCs may have the ability to reduce the side effects caused by chemotherapy. The monoclonal antibody in ADC delivers drugs directly to the tumor site to improve the targeted killing effect and reduce off-target toxicity^2^.

### The use of ADC in solid tumors

ADCs have been applied to some solid tumors and have demonstrated good efficacy in cancers including breast, cervical, gastric, and ovarian cancers. A phase 2 study (NCT03438396) was done to evaluate the efficacy and safety of tisotumab vedotin, an ADC, in patients with cervical cancer. And tisotumab vedotin showed clinically meaningful and durable antitumor activity with a manageable and tolerable safety profile^3^. What is more, another phase 2 study (NCT03505710) in which trastuzumab deruxtecan (another type of ADC) was administered to patients who had NSCLC found that trastuzumab deruxtecan showed durable anticancer activity in patients with previously treated HER2-mutant NSCLC^4^. Moreover, the field of breast cancer and gastric cancer has also seen the excellent efficacy of ADC drugs, such as Trastuzumab Emtansine and Trastuzumab deruxtecan, in clinical use^5^.

### The use of ADC in pancreatic cancer

Despite the great success of ADCs in the treatment of some solid tumors, they have not yet demonstrated the same potential in clinical trials for pancreatic cancer. According to www.clinicaltrials.gov, by the end of 2023, more than a dozen ADCs for pancreatic cancer are in various stages of clinical studies, yet only a few results have been published. These are enough to see that the aggressiveness and complexity of pancreatic cancer pose many challenges for ADC’s development.

Even so, based on the current clinical data, ADCs still have great potential in the pancreatic cancer field. Sacituzumab govitecan (IMMU-132) is an ADC. Clinically relevant dosing schemes of IMMU-132 administered in mice bearing human pancreatic cancer xenografts demonstrate significant antitumor effects in models. Current Phase I/II clinical trials (NCT01631552) confirm anticancer activity of IMMU-132 in pancreatic cancer patients^6^. Furthermore, some researchers developed an ADC, hSD5-vedotin, specifically targeting EphA2 in pancreatic cancer cells. In a pancreatic cancer xenograft animal model, hSD5-vedotin showcased the potential to suppress tumor growth entirely. Notably, potential immune resistance responses were also observed in recurrent pancreatic cancer tumors^7^. In addition, MUC1 is also an overexpressed target in pancreatic cancer. Researchers generated the ADC by conjugating HzMUC1 with MMAE, and examined the efficacy of HzMUC1-MMAE against the MUC1-positive pancreatic cancer in vitro and in vivo. Results indicate that HzMUC1-ADC is a promising novel targeted therapy for pancreatic cancer^8^.

### Extreme shortage of drugs in the field of pancreatic cancer

In short, pancreatic cancer is now in a similar, if not worse, position than gastric cancer in terms of drug therapy. As Vincent J. Picozzi *et al*. mentioned in their article, monoclonal antibody drugs are more often used for first-line treatment. However, the use of immunotherapy in second-line and third-line treatment of pancreatic cancer was not mentioned in detail. Take the current use of ADCs in gastric cancer, for example, many options for third-line treatment of advanced GC or gastroesophageal junction carcinoma (GEJC) have been developed. Therapies including immunotherapy (nivolumab), chemotherapy (irinotecan, FTD/TPI), targeted therapy (apatinib), and ADC have shown to increase the survival rates in patients. It is noteworthy that researchers found that ADC may be the best option for the overall GC population for third-line therapy, followed by the monoclonal antibody, navumab^9^. And could this result also happen in the field of pancreatic cancer treatment?

## ADC is not a level-up version of monoclonal antibody

Due to the outstanding performance of ADCs drugs in recent years, many researchers now consider that further development of ADCs may replace the current position of monoclonal antibodies in clinical treatment. This is because from the structure of ADCs, ADCs are more like a combination of chemotherapy and immunotherapy, which can play the roles of both chemotherapy and immunotherapy.

**Figure 1:**
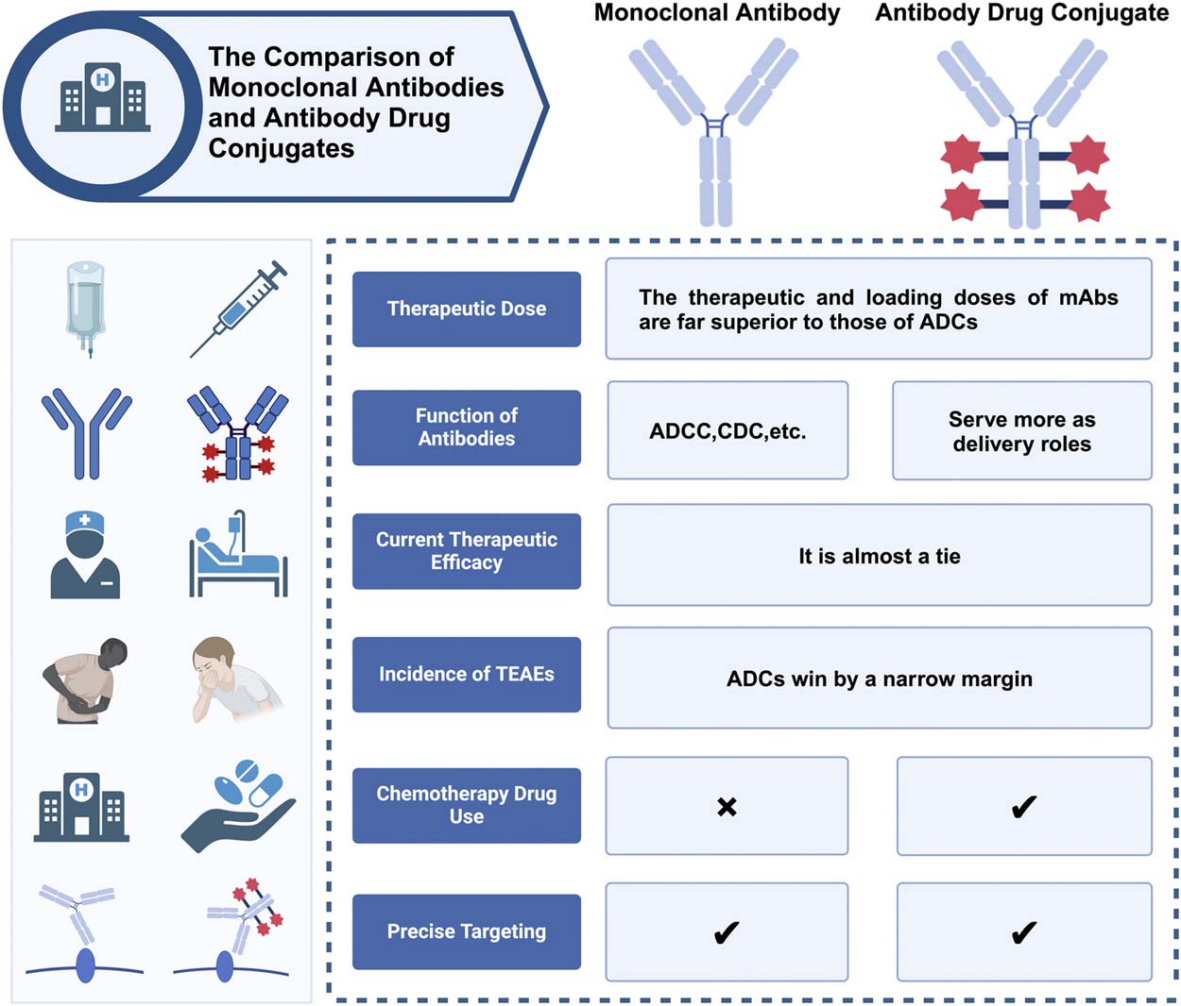
The comparison of monoclonal antibodies and antibody drug conjugates.

However, from the perspective of immunotherapy, ADCs do not, to a certain extent, fully accomplish the work undertaken by antibodies and induce the complete biological effect of antibodies. What is more, ADCs do not complete the same work that antibodies do, eliciting the full biological effect of the antibody. In fact, ADCs are more like a ‘navigator’ for the chemotherapeutic agent. In other words, ADCs are more like upgrades to chemotherapeutic drugs than upgrades to monoclonal antibodies. The comparison of monoclonal antibodies and ADCs has also shown in Figure 1.

### Differences in dosage lead to differences in effectiveness

Differences in dosage prevent ADCs from exerting the same efficacy as monotherapy drugs. The different dosages used for ADCs compared to monoclonal antibody drugs may have contributed to the fact that ADCs do not fully utilize the immunological effects of their antibodies.

In terms of dosage, Zolbetuximab, a monoclonal drug, whose clinical use dosage regimen is that patients receive 800 mg/m^2^ of Zolbetuximab as a loading dose, followed by 600 mg/m^2^ of Zolbetuximab every 3 weeks^10^. Compared to Zolbetuximab’s loading dose of ~20 mg/kg, ADC’s is almost unattainable (approximately 3 mg/kg)^11^. Antibodies mainly exert effector functions such as ADCC, CDC, and so on. Apparently low doses of ADCs can hardly perform such functions.

### Antibodies in ADCs assume different roles from those of monoclonal antibodies

Antibodies in ADCs act more as delivery roles and take on more of a targeted delivery role. Antibodies alone in ADCs are difficult to demonstrate biological activity in immunodeficient mouse models at ADC’s clinical doses. At the same time after coupling ADC whether sulfhydryl coupling or 297 sugar fixed-point coupling will weaken the ADCC effect to a certain extent, to some degree, it may lead to the weakening of the antibody activity in the ADC. Therefore, DS-8201(ADC) did not show the cardiotoxicity and myocarditis caused by trastuzumab. From this point of view, the use of ADC compared to the use of monoclonal antibody may reduce a certain toxic effect.

### In terms of current therapeutic efficacy, ADC is not an upgrade from monoclonal antibody

Many people currently view ADCs as an upgrade to monoclonal antibody drugs, and even believe that ADCs will replace existing monoclonal antibody drugs in the not-too-distant future. However, according to the available clinical data to compare the therapeutic effects of the two, ADC has not become the monoclonal antibody 2.0.

In order to compare the efficacy of the two drugs more objectively, the latest clinical trial results of monoclonal antibody and ADC for the same target and the same cancer type were selected. After filtering, we have selected VYLOY and CMG901 to illustrate the description. VYLOY is a CLDN18.2 monoclonal antibody for patients with gastric cancer. The Phase 3 SPOTLIGHT and GLOW clinical trials for first-line treatment in patients with gastric or gastroesophageal junction (GEJ) adenocarcinoma whose tumors were CLDN18.2 positive evaluated VYLOY plus different chemotherapies compared to placebo plus chemotherapies^12,13^. CMG901, is a CLDN18.2-targeted ADC carrying MMAE. A phase 1 trial of CMG901 in patients with advanced gastric/gastroesophageal junction cancer (NCT04805307) has announced that it has demonstrated potent antitumor activity in preclinical studies^11^.

First, we compared the efficacy of monoclonal antibodies with ADCs by using ORR as a criterion. We found that monoclonal antibody drugs are at the leading place. Results from GLOW showed that in patients with measurable disease, ORR (95% CI) was 53.8% (46.58−60.99) versus 48.8% (41.76−55.84) in VYLOY versus placebo arm^14^. However, CMG901 performed less well. Of 89 evaluable (≥1 post-treatment scan) CLDN18.2-positive patients, confirmed ORR was 32.6%^11^.

In addition, we compared the incidence of therapeutic emergency adverse events (TEAEs) when using monoclonal antibodies versus ADCs. There was in fact little difference between them. The most frequent treatment-emergent adverse events (TEAEs) ≥20% for VYLOY in combination with mFOLFOX6 or CAPOX were nausea, vomiting, decreased appetite, neutropenia, and decreased weight^12,13^. To be more specific, most common TEAEs with VYLOY + mFOLFOX6 were nausea (82.4 vs. 60.8% in VYLOY vs. placebo arms), vomiting (67.4 vs. 35.6%), and decreased appetite (47.0 vs. 33.5%); the incidences of serious TEAEs were similar between both arms (44.8 vs. 43.5%). When it comes to CMG901 used in the clinical trial, most common TEAEs were anemia (62.8%), vomiting (57.5%), and hypoalbuminemia (57.5%). Neutrophil count decreased (18.6%) and anemia (13.3%) were the most frequent grade ≥3 TEAEs^11^.

Above all, according to the results of available clinical trials, ADCs do not show a huge advantage in terms of efficacy over monoclonal antibodies. Therefore, it is debatable that ADCs are an upgrade to monoclonal antibody drugs.

## The future of ADC drugs in pancreatic cancer

### Potential to be a third-line and above treatment option for pancreatic cancer

Due to the extreme shortage of drugs in the field of pancreatic cancer, as long as the efficacy of ADCs outperforms that of last-line chemotherapy, there are more chances that ADC replaces the original form of pancreatic cancer treatment. It is frustrating that, to date, there are no clinical data demonstrating the efficacy of an ADC in pancreatic cancer patients who have received two or more prior chemotherapy treatments. However, since gastric and pancreatic cancers share many common therapeutic targets, there is already a new ADC in the field of gastric cancer, T-DXd, which has shown a significantly higher objective response rate and a longer overall survival and, thus, was approved in Japan^15^. So perhaps in the near future, new ADCs for the pancreatic cancer field will also be produced. For now, based on the results of available clinical trials, the outlook for the use of ADCs for second-line and third-line treatment of pancreatic cancer is very promising.

### Use of combination therapy and ICI may help boost ADC outcomes

The efficacy of ADCs as a single agent is still limited, which is likely due to several factors which include the characteristics of the ADC itself such as choice of tumor antigen, linker molecule or payload^2^.

Combination therapies may be able to solve the dilemma. The combination of gemcitabine with ADCs has been investigated in a number of preclinical studies^16–18^. TR1801ADC, an ADC which targets the tumor antigen MET and is conjugated to the PBD toxin, tesirine. Researchers found that TR1801-ADC was able to synergize with gemcitabine and improve tumor response in gemcitabine-resistant PDX tumors^17^. Similarly, MLN0264 an anti-GCC targeting ADC showed superior tumor growth inhibition when combined with gemcitabine, compared to either therapy alone in pancreatic PDX models^18^.

Furthermore, the combination of ADCs and immune checkpoint inhibitors (ICIs) may reverse the limited efficacy of ADCs used as single agents. MGC018 is a duocarmycin-based ADC that targets B7-H3 in pancreatic cancer. Preclinical studies showed the combination of MGC018 with anti-PD-1 checkpoint inhibition being synergistic^19^. However, at present, MGC018 is still being evaluated in a phase I clinical trial (NCT05293496). What is more, Disitamab vedotin (RC48) is an HER2-directed ADC. Human PD-1 transgenic mice were used to analyze the in vivo antitumor effects of the ADC and its combination therapy with PD-1/PD-L1 antibody. The combination of RC48 and PD-1/PD-L1 immune checkpoint inhibition significantly enhanced tumor suppression and antitumor immunity. Tumor rejection in the synergistic groups was accompanied by massive T cell infiltration and immune marker activation. Furthermore, the combination therapy promoted immunological memory formation in the tumor eradication animals, protecting them from tumor rechallenge^20^.

### Searching for better drug targets and better cytotoxic drug in pancreatic cancer

Precise targeting of tumor antigens is critical to the success of ADCs, and the optimal target for pancreatic cancer therapy which directed against pancreatic cancer is inconclusive.

A key factor in obtaining effective ADCs is the selection of appropriate tumor-specific antigens. Current promising ADC targets include EGFR, Mesothelin, CEA, Trop2, CD142, HER3, CD70, MUC1, ICAM1, CD71, GPC1, SLC44A4, DR5, etc.^2^. Of these, CLDN18.2 is more prevalent these days. Human CLDN18.2 is highly expressed in a significant proportion of gastric and pancreatic adenocarcinomas, while normal tissue expression is limited to the epithelium of the stomach. However, there are still many ADCs for different targets in pancreatic cancer in clinical trials. At the same time, the existing marketed drugs for each target are constantly being updated and upgraded. Moreover, for each target, at least two forms of drugs are needed, one in the form of monoclonal antibodies and the other in the form of ADCs.

Researchers can also enhance the potency of ADC drugs by replacing cytotoxic drugs from existing ADC drugs. As is said at the beginning, ADC consists of a monoclonal antibody, a linker and a cytotoxic drug. The potency of the drug can also be enhanced by changing the type of cytotoxic drug linked to the antibody in the ADC. There is a randomized, open-label, phase 3 trial comparing trastuzumab deruxtecan and trastuzumab emtansine’s efficacy in patients with HER2-positive metastatic breast cancer^21^. These two ADCs share the same antibody, but the cytotoxic drug attached to their respective antibodies is different. This trial found that trastuzumab deruxtecan showed a significant improvement in overall survival versus trastuzumab emtansine in patients with HER2-positive metastatic breast cancer, as well as the longest reported median progression free survival^21^.

### ADCs have potential as neoadjuvant therapy for pancreatic cancer

In other cancer types, researchers are also engaged in exploring the possibility of ADC as a neoadjuvant therapy. However, in the field of pancreatic cancer, there are no relevant clinical data to confirm its feasibility. The HER2 targeting ADC trastuzumab emtansine, trastuzumab deruxtecan, Trop-2 targeting ADC sacituzumab govitecan, Brentuximab vedotin and enfortumab vedotin has shown great efficacy as neoadjuvant chemotherapy in breast cancer, lymphoma, and invasive urothelial cancer^22–27^. The success of ADCs as neoadjuvant therapy in other cancers could provide a new reference for ADCs as neoadjuvant therapy in pancreatic cancer.

## Ethical approval

There are no relevant trials requiring ethical approval in our article.

## Consent

There are no relevant trials in our article.

## Source of funding

This work was supported by Postdoctoral Fellowship Program of CPSF (No. GZB20230481), Post-Doctor Research Project, West China Hospital, Sichuan University (No. 2024HXBH149, No. 2024HXBH006), National Natural Science Foundation of China (No. 82303773, No. 82303772, No. 82204490), Natural Science Foundation of Sichuan Province (No. 2023NSFSC1885, No. 2024NSFSC1908), Key Research and Development Program of Sichuan Province (No. 23ZDYF2836).We express our thanks to BioRender.com for creating Figure 1.

## Author contribution

Y.Y.: writing – original draft and visualization; Y.Z.: writing – original draft and writing – review and editing; X.S.: original draft and visualization; A.Z.: conceptualization and supervision; Y.W.: conceptualization, writing – review and editing, and supervision.

## Conflicts of interest disclosure

There are no conflicts of interest.

## Research registration unique identifying number (UIN)

There are no relevant trials in our article.

## Guarantor

All the authors of this paper accept full responsibility for the work and/or the conduct of the study, have access to the data, and control the decision to publish.

## Data availability statement

No primary data were generated and reported in this manuscript. Therefore, data have not become available to any academic repository.

## Provenance and peer review

Our paper was not invited.
